# Wilms’ tumor susceptibility: possible involvement of *FOXP3* and *CXCL12* genes

**DOI:** 10.1186/s40348-016-0064-4

**Published:** 2016-11-10

**Authors:** Patricia Midori Murobushi Ozawa, Carolina Batista Ariza, Roberta Losi-Guembarovski, Alda Losi Guembarovski, Carlos Eduardo Coral de Oliveira, Bruna Karina Banin-Hirata, Marina Okuyama Kishima, Diego Lima Petenuci, Maria Angelica Ehara Watanabe

**Affiliations:** 1 Department of Pathological Sciences, Laboratory of Study and Application of DNA Polymorphisms, Biological Sciences Center, State University of Londrina, Londrina, PR Brazil; 2 Department of Pathology, Clinical Analysis and Toxicology, Health Sciences Center, State University of Londrina, Londrina, PR Brazil

**Keywords:** Wilms’ tumor, FOXP3, CXCL12, Genetic polymorphism

## Abstract

**Background:**

Wilms’ tumor is an embryonal neoplasm of the kidney that accounts for approximately 6 % of all childhood tumors. The chemokine CXCL12 (C-X-C chemokine ligand 12) and its ligand CXCR4 (C-X-C chemokine receptor type 4) are involved in the development of several organs, including the kidney, and are also associated with tumor growth and metastasis. FOXP3 (forkhead transcription factor 3) was initially described as a marker for regulatory T cells; however, its expression in several types of tumor cells has already been described and may have prognostic significance. The aim of the present study was to analyze rs3761548 and rs2232365 *FOXP3* polymorphisms, as well as evaluate rs1801157 *CXCL12* polymorphism in Wilms’ tumor samples.

**Methods:**

Polymorphisms were evaluated in 32 patients and 78 neoplasia-free controls. Genotypes of rs1801157 were determined using PCR-restriction fragment length polymorphism (PCR-RFLP) method, and genotypes of rs2232365 and rs3761548 were determined using allele-specific PCR (AS-PCR).

**Results:**

The case-control study indicated a significant association for allele A carriers of rs1801157 polymorphism in relation to Wilms’ tumor susceptibility (OR = 5.261; 95 % CI 2.156 to 12.84; *p* = 0.0002). The opposite was observed in male carriers of G allele for rs2232365 polymorphism (OR 0.1164; 95 % CI 0.0227 to 0.5954; *p* = 0.0091) or when male and female subjects were analyzed (OR = 0.1304; 95 % CI 0.05013 to 0.3394; *p* < 0.0001).

**Conclusions:**

All in all, these markers may contribute to this neoplasia susceptibility and progression; however, further studies are needed to real clarify their role in Wilms’ tumor pathogenesis.

## Background

Childhood cancers differ from adult malignant neoplasia in several aspects, such as in primary and histological origins and, also, in clinical outcomes, suggesting they have to be studied independently from adult cancer [1]. Besides, their early onset suggest a low exposition to risk factors, indicating that genetic alterations may have major influences in childhood tumor development [2].

The Wilms’ tumor (WT) develops from nephroblastic remnants, and it is characterized as an embryonal tumor, composed of persistent blastema, dysplastic tubules, and supporting mesenchyme or stroma [3]. It accounts for approximately 6 % of all childhood tumors [4], and its incidence corresponds to 1 in 10,000 children. The majority of WT are usually unilateral and sporadic, with only 1 % considered hereditary [5].

The tumor microenvironment is composed of neoplastic and stromal cells and a great number of immune cells. Interactions among tumor microenvironment components are an emerging issue in tumor progression, influencing growth, invasiveness, and metastatic process [6]. Understanding these complex networks is extremely important for prognostic markers discovery and development of new therapeutic strategies [7].

Chemokines play a major role in several homeostatic [8], pathological [9], and developmental processes [10]. Among them, C-X-C chemokine ligand 12 (CXCL12) and its receptor C-X-C chemokine receptor type 4 (CXCR4) seem to be involved in the development of several organs [11, 12], including kidney [13], and they are also related to tumor growth [14] and metastatic process in many types of cancer [15]. Some authors have investigated polymorphisms of *CXCL12* in disease pathogenesis, including cancer, [16] but its value as a susceptibility marker is not well determined.

The forkhead box protein 3 (FOXP3) is a transcription factor that has a fundamental role on the regulation and development of the immune system [17, 18]. Although it was first described as restricted to hematopoietic lineages, recent studies have shown FOXP3 expression in several tissues, including tumor cells [19–21], and it has also been suggested a nuclear or cytoplasmic localization, which can be related with patient prognosis [22].

Genetic analysis of some diseases like psoriasis [23] and breast cancer [24] showed significant association with the single nucleotide polymorphisms (SNP) rs3761548 (−3279 C/A) and rs2232365 (−924 A/G) of *FOXP3* gene [25]. The study of these allelic variants can elucidate the role of such polymorphisms in several pathologies, including cancer, concerning to susceptibility, and prognosis.

Recently, a crosstalk between FOXP3 and CXCR4 has been described by Douglass et al. [26]. They demonstrated that downregulated FOXP3 cells have increased CXCR4 expression, and their migration toward CXCL12 gradient is higher when compared with cells who expressed higher FOXP3 levels.

The present study aimed to analyze two polymorphisms in *FOXP3* and one polymorphism in *CXCL12* in WT samples, in a search for new possible molecular markers to this childhood neoplasia.

## Methods

### Human subjects

A total of 32 paraffin-embedded samples containing normal and tumor tissues was obtained at University Hospital of the State University of Londrina, Londrina, Paraná, Brazil. Clinical data presented (age, tumor size, and gender) were obtained from clinical pathological reports. For control group, blood samples from 78 neoplasia-free individuals were collected at the same region, with an informed consent signed by their parents. This study was conducted following approval from the Human Ethics Committee of State University of Londrina (CEP/UEL 189/2013 – CAAE 17123113400005231), which was in compliance with the declaration of Helsinki.

### DNA extraction

Genomic DNA was isolated from formalin-fixed paraffin-embedded samples, according to innuPREP DNA Mini Kit (Analytik Jena AG, Jena, Germany) protocol, following manufacturer’s instructions. For neoplasia-free control group, DNA was obtained from peripheral blood white cells using the extraction kit Mini Spin (Biometrix, Curitiba, PR, Brazil), according to manufacturer’s instructions. All DNA samples were quantified in NanoDrop 2000® (NanoDrop Technologies, Wilmington, DE, USA).

### Genotyping of CXCL12 and FOXP3 polymorphisms

Genotypes of rs1801157 were determined using polymerase chain reaction (PCR)-restriction fragment length polymorphism (PCR-RFLP) method, and genotypes of rs2232365 and rs3761548 were determined using allele-specific PCR (AS-PCR) [23, 27]. Reactions were performed with 100 ng of genomic DNA, 100 μM dNTP, 150 ρM of each primer (Table 1), MgCl_2_ 1.5 mM, buffer 10 %, and 1.25 units of Taq DNA polymerase (Invitrogen, Carlsbad, CA, USA), in a thermocycler A200 Gradient Thermal Cycler (LongGene, Hangzhou, China). PCR products of CXCL12 were subjected to restriction digestion by incubation with *Msp*I (10 U) (Promega, Madison, WI, USA) during 4 h at 37 °C. All PCR products were analyzed by electrophoresis on polyacrylamide gel (10 %) and detected using a silver staining method.

**Table 1:** Primer sequences of *FOXP3* and *CXCL12* genes

		Primer sequence	PCR product
RFLP-PCR	rs1801157	5′-CAGTCAACCTGGGCAAAGCC-3′	293 bp
	*CXCL12*	5′-CCTGAGAGTCCTTTTGCGGG-3′	
AS-PCR		5′-CTGGCTCTCTCCCCAACTGA-3′	Allele A 334 bp
	rs3761548	5′-ACAGAGCCCATCATCAGACTCTCTA-3′	
	*FOXP3*	5′-TGGCTCTCTCCCCAACTGC-3′	Allele C 333 bp
		5′-ACAGAGCCCATCATCAGACTCTCTA-3′	
		5′-CCCAGCTCAAGAGACCCCA-3′	Allele A 442 bp
	rs2232365	5′-GGGCTAGTGAGGAGGCTATTGTAAC-3′	
	*FOXP3*	5′-CCAGCTCAAGAGACCCCG-3′	Allele G 427 bp
		5′-GCTATTGTAACAGTCCTGGCAAGTG-3′	
Sequencing	rs3761548	5′-TCTCCGTGCTCAGTGTAGAA-3′	330 bp
	*FOXP3*	5′-AACTAGGCCTCCTGACCTATG-3′	
	rs2232365	5′-AGAAGGAGTGGGCATTTGAG-3′	284 bp
	*FOXP3*	5′-GCAGGTGTAGATAGACATGAAGAG-3′	

AS-PCR for FOXP3 polymorphisms were confirmed by randomly sequencing in 15 % of the samples. After amplification, PCR products were purified using PureLink™ PCR Purification Kit (Invitrogen), following manufacturer instructions. The sequencing reaction was performed using BigDye® Terminator v3.1 Cycle Sequencing Kit (Applied Biosystems®, Foster City, CA, USA), 50 ng of DNA template and 5 ρM of primer (forward or reverse) in a final volume of 10 μl. PCR conditions were as follows: 5 min denaturing at 95 °C, 30 cycles of 20 s at 95 °C, 20 s at 50 °C, and 1 min at 60 °C. The amplicons were sequenced in a 24-capillary 3500xl Genetic Analyzer (Applied Biosystems).

### Statistical analysis

Case-control study association was assessed through odds ratio (OR) analysis, adopting 95 % confidence interval (CI), and Fisher’s exact test, performed using Prism 6 for Windows (GraphPad Software, San Diego, CA, USA). Since *FOXP3* gene in humans is located in the p-arm of the X-chromosome at Xp.11.23, polymorphism analysis was performed separately for genders. *p* value <0.05 was considered statistically significant.

## Results

This retrospective study evaluated 32 tissue samples of pathologically confirmed patients diagnosed with WT between January 1990 and December 2013. The mean age at diagnosis was 45 months (range 1 year–13 years), and more than 76 % of cases diagnosed before the age of 5 years old, which is in accordance with literature data [5].

Nineteen (59.37 %) tumoral tissues were obtained from female patients and 13 (40.63 %) were from male patients. Information regarding tumor size was recovered from 25 (78.12 %) samples, once records available through the hospital were not necessarily historically complete or present. This parameter ranged from 6 to 20.5 cm, with an average of 8 cm, which was used to perform the analysis in relation to genetic variants. From 25 samples, seven (28 %) had tumor size less than or equal to 8 cm and 18 (72 %) had tumor size larger than 8 cm.

Likewise, not all data regarding capsular invasion (*n* = 17), metastasis (*n* = 11), lymph node involvement (*n* = 18), and staging (*n* = 22) were available. Such parameters are summarized in Table 2.

**Table 2:** Histopathological parameters of Wilms’ tumor samples

Capsular invasion	Presence	11
	Absence	6
Metastasis	Presence	7
	Absence	4
Lymph node involvement	Presence	5
	Absence	13
Tumor staging	I	6
	II	6
	III	3
	IV	6
	V	1

Considering WT histology, ten samples were classified as blastemal, six samples presented mixed type, four samples were epithelial, and two monophasic tumors (cells with vesicular nuclei, visible nucleoli, and acidic cytoplasm). It has not been possible to obtain ten samples data regarding tumor histological classification. The preoperative chemotherapy was used in four samples.

In the control group, children and adolescents (age average 12 years old) were included according to negative hematological, biochemical, and serological tests for infectious or chronic diseases and consisted of 37 (47.4 %) females and 41 (52.6 %) males.

### CXCL12 genetic polymorphism

**Fig. 1:**
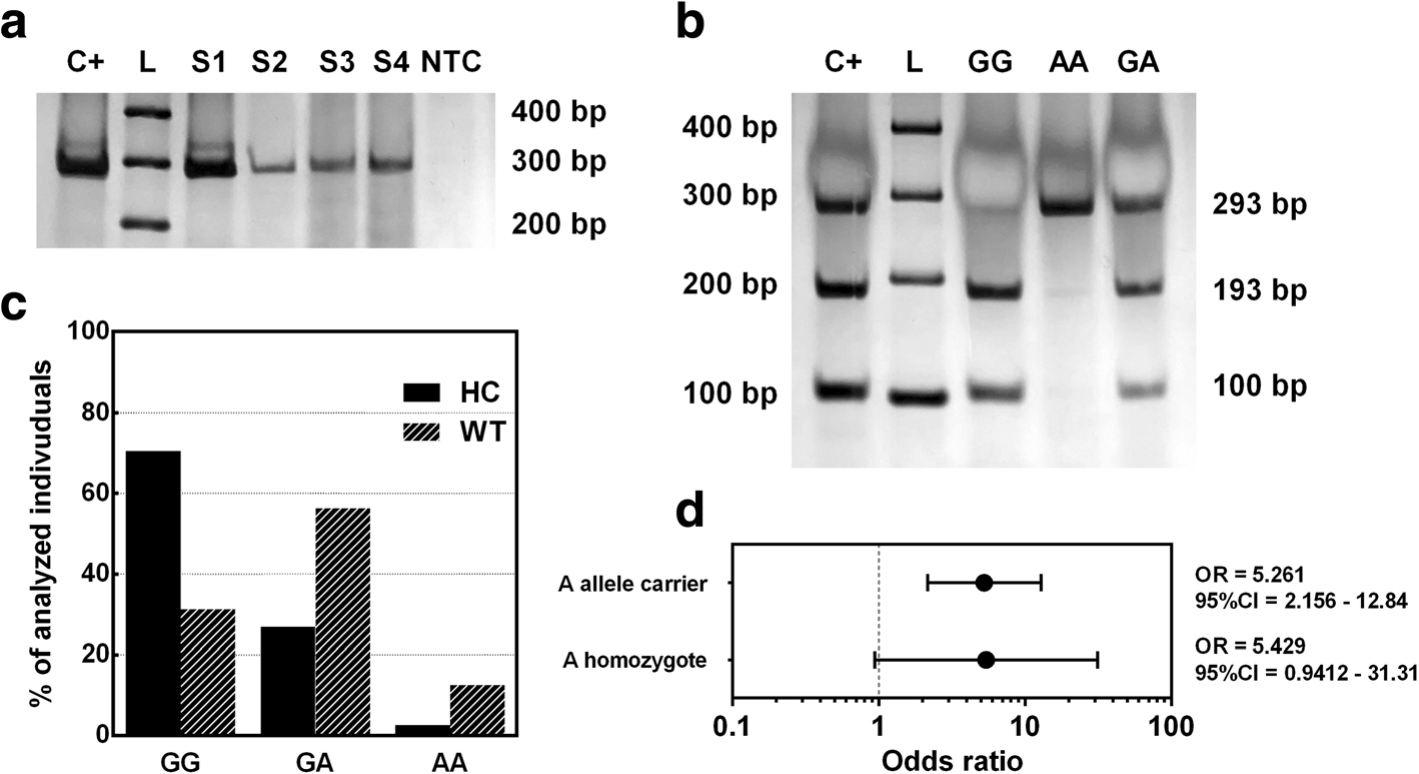
*CXCL12* rs1801157 polymorphism. **a** Electrophoretic profile of *CXCL12* polymorphism. *C+* positive control, *L* ladder 100 bp, *S1, S2, S3, S4* samples, *NTC* no template control. **b** Electrophoretic profile of *MspI* treatment products. *C+* positive control for both alleles (GA), *L* ladder 100 bp, *GG* wild-type homozygote, *AA* mutant homozygote, *GA* heterozygote. **c** Genotype distribution of healthy control (*HC*) and Wilms’ tumor (*WT*) individuals. **d** Odds ratio analysis

The electrophoretic profile of rs1801157 *CXCL12* polymorphism is represented in Fig. 1a, b. Figure 1a shows the PCR fragment of 293 bp and the *Msp*I enzyme cut amplicons without the polymorphic variant. Thus, the *CXCL12* GG genotype produces 100 and 193 bp products; the AA genotype produces a 293 bp, and the heterozygote genotype GA produces three distinct fragments (Fig. 1b).

The genotype frequency observed for *CXCL12* polymorphism for WT patients and controls is represented in Fig. 1c, in which 78 % (22/32) are carriers of the variant allele A. The case-control study indicated a strong positive association of more than fivefold, for A allele carriers, with WT susceptibility (*p* = 0.0002) (Fig. 1d).

### FOXP3 genetic polymorphisms

**Fig. 2:**
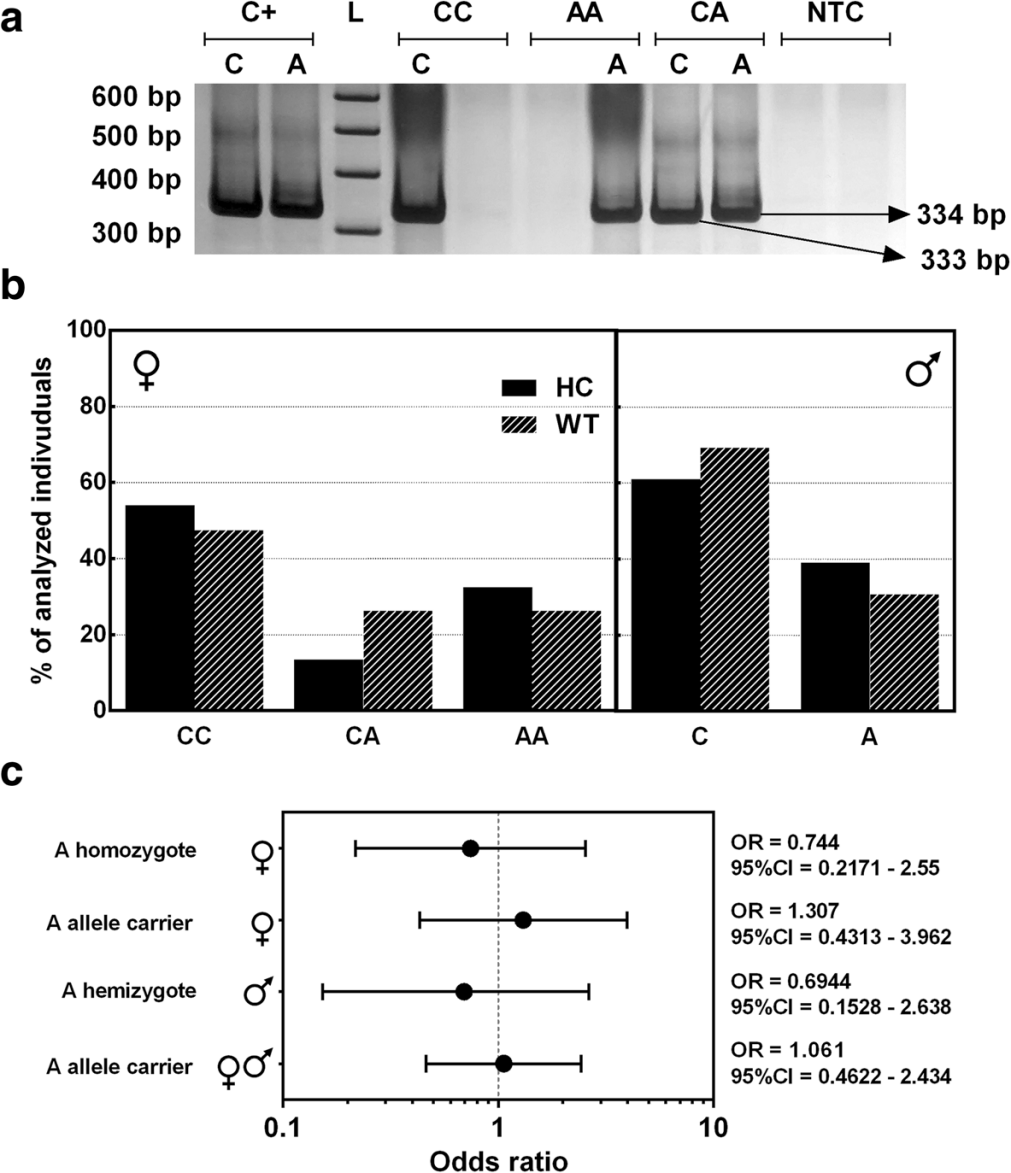
Analysis of *FOXP3* rs3761548 polymorphism. **a** Electrophoretic profile. *C+* positive control for allele C and A, *L* ladder 100 bp, *CC* wild-type homozygote genotype, *AA* variant homozygote, *CA* heterozygote genotype, *NTC* no template control. **b** Genotype frequency of healthy control (*HC*) and Wilms’ tumor (*WT*) individuals, in female individuals (*left*) and male individuals (*right*). **c** Odds ratio analysis

In the present study, we also investigated two single nucleotide polymorphisms (SNPs) on the promoter region of *FOXP3* gene. The electrophoretic profile for polymorphism rs3761548 can be observed in Fig. 2a. As illustrated in Fig. 2b—left panel, genotype frequencies of rs3761548 for female patients and controls were as follows: 47.4 % (9/19) and 54.1 % (20/37) for CC homozygote, 26.3 % (5/19) and 13.5 % (5/37) for CA heterozygote, and 26.3 % (5/19) and 32.4 % (12/37) for AA homozygote, respectively. Male patients and control genotype frequencies for this same polymorphism were as follows: 69.2 % (9/13) and 61.0 % (25/41) for C hemizygote and 30.8 % (4/13) and 39.0 % (16/41) for A hemizygote (Fig. 2b—right panel).

In this work, no significant association was observed for AA homozygotes or A allele carriers in relation to WT susceptibility (*p* > 0.05; Fig. 2c). Moreover, A allelic frequency of rs3761548 was higher in WT patients (62.5 %) than in the control group (58.97 %).

**Fig. 3:**
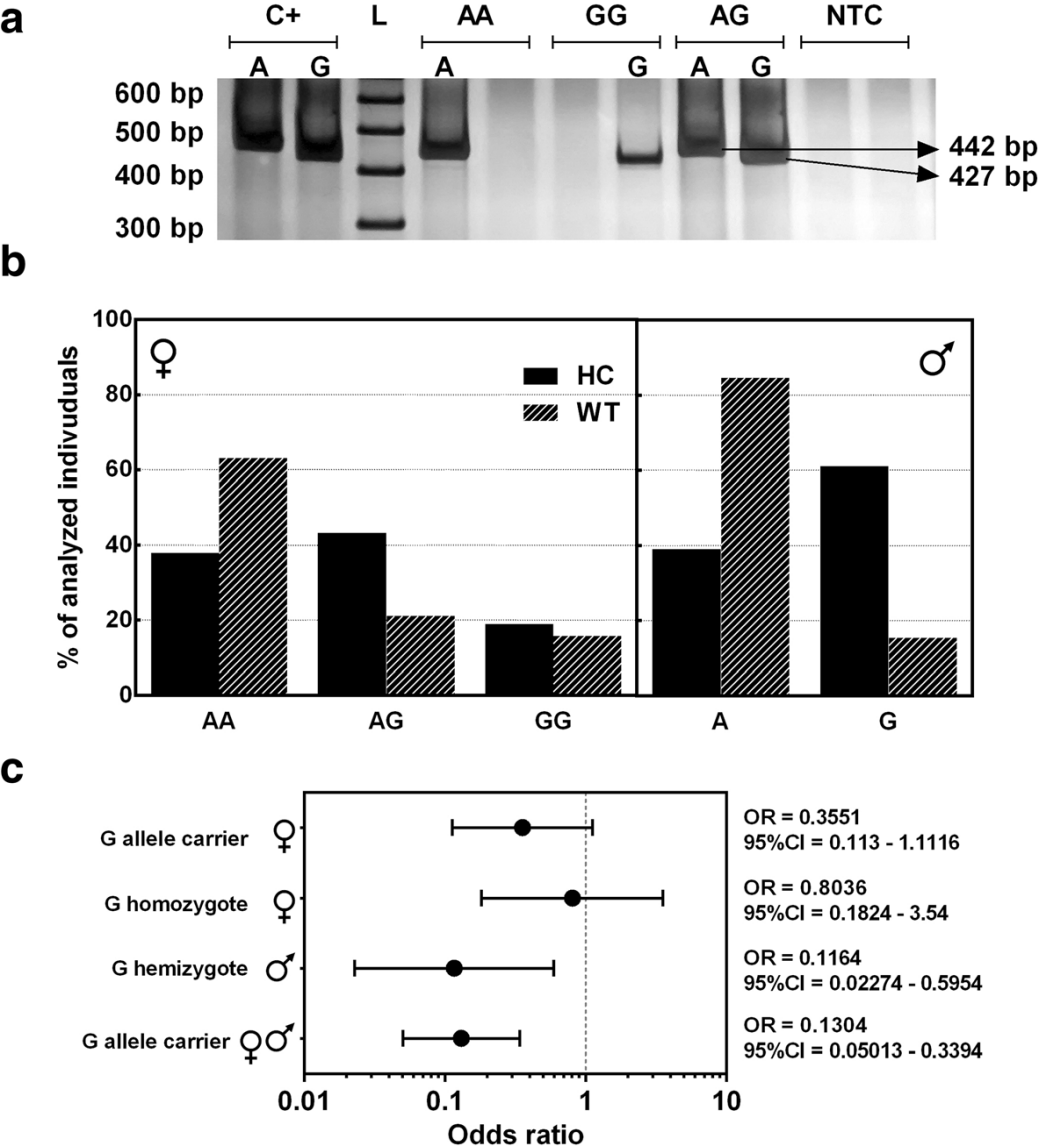
Analysis of *FOXP3* rs2232365 polymorphism. **a** Electrophoretic profile. *C+* positive control for allele A and G, *L* ladder 100 bp, *AA* wild-type homozygote genotype, *GG* mutant homozygote genotype, *AG* heterozygote genotype, *NTC* no template control. **b** Genotype frequency of healthy control (*HC*) and Wilms’ tumor (*WT*) individuals, in female individuals (*left*) and male individuals (*right*). **c** Odds ratio analysis

Regarding the *FOXP3* rs2232365 polymorphism, electrophoretic profile is represented in Fig. 3a. The genotype frequency observed was 63.2 % (12/19) and 37.8 % (14/37) for AA homozygotes, 21.0 % (4/19) and 43.3 % (16/37) for AG heterozygotes, and 15.8 % (3/19) and 18.9 % (7/37) for GG homozygote, for female patients and controls, respectively. The genotype frequencies for male patients and controls were as follows, respectively: 85.6 % (11/13) and 39.0 % (16/41) for A hemizygotes and 15.4 % (2/13) and 61.0 % (25/41) for G hemizygotes (Fig. 3b). Some products of AS-PCR for rs3761548 and rs2232365 were confirmed by direct automated sequencing of PCR products using BigDye terminator chemistry kit and 3500 Genetic Analyzer.

The case-control study indicated that G allele carriers of *FOXP3* polymorphism rs2232365 were negatively associated with WT susceptibility, comparing male individuals (OR 0.1164; 95 % CI 0.0227 to 0.5954; *p* = 0.0091), and when male and female subjects were analyzed together (OR = 0.1304; 95 % CI 0.05013 to 0.3394; *p* < 0.0001) (Fig. 3c).

## Discussion

Studies have shown that WT cells express markers of early kidney development [28, 29]. In addition, several studies have highlighted the presence and importance of CXCL12 and CXCR4 during kidney maturation [13, 30–32]. In this context, genotype frequencies of *CXCL12* polymorphism rs1801157 have been investigated in order to address its possible role in tumor pathogenesis in different conditions, including acute lymphoblastic leukemia [16], chronic myelogenous leukemia [33], breast cancer [34, 35], and Hodgkin’s lymphoma and non-Hodgkin’s lymphoma [35]. However, there was no study in literature indicating the frequency of this polymorphism in WT patients. In the present case-control study, it was verified a strong positive association for A allele carriers and WT susceptibility (Fig. 1).

Polymorphisms in regulatory regions can change protein expression and may be associated with susceptibility to certain diseases [27]. In fact, the rs1801157 polymorphism is located at a regulatory region of *CXCL12*; however, there are conflicting results about the influence of this polymorphism in protein expression. Some studies have shown that A allele carriers have increased CXCL12 protein levels [27, 36]; on the other hand, de Oliveira et al. [34] observed that A allele carriers had low levels of CXCL12 messenger RNA (mRNA) compared to GG genotype.

These contrast CXCL12 expression patterns might represent different techniques (serum ELISA, mRNA expression, blot analysis) or biological samples tested (peripheral blood, cultured cells). Moreover, prospective studies should be developed in order to provide rational conclusions on how CXCL12 rs1801157 genotypes would influence gene transcription and/or translation.

It is known that spatial and temporal relationship between CXCL12- and CXCR4-positive cells are required for a regular kidney development [13]. In light of our results, the authors would suggest that A allele carriers, which may express altered CXCL12 levels, could be more susceptible to kidney development disruption.

*FOXP3* is an X-linked gene that encodes a transcription factor, which is essential in CD4^+^CD25^+^FOXP3 regulatory T (Treg) cells [37]. Treg cells may contribute to tumorigenesis by suppressing immune responses from host, and mutations of this gene have already been reported in cancer patients [38]. To date, there are no studies investigating *FOXP3* polymorphisms in WT patients. Regarding the abovementioned, investigation of possible association of *FOXP3* genetic variants in WT may shed light on the molecular pathogenesis of this neoplasia, opening up new paths to screening susceptible individuals.

Although AA homozygotes for rs3761548 *FOXP3* polymorphism have been considered susceptible to breast neoplasia [24], no significant association was observed for AA homozygotes or A allele carriers in relation to WT susceptibility (Fig. 2c). Furthermore, allelic distribution of rs3761548 A allele in WT patients was slightly different from that in the control group. Concerning *FOXP3* rs2232365 polymorphism, the case-control study indicated that G allele is negatively associated with WT susceptibility in male individuals and when males and females subjects were analyzed together (Fig. 3c).

The *FOXP3* rs2232365 polymorphism is located within a putative DNA-binding site of another transcription factor, GATA-3, that directly regulates FOXP3 expression, in addition to controlling Treg cell function via interaction with the regulatory regions of the *FOXP3* locus. GATA-3 is essential to Th2 immune response [39] and can only bind the *FOXP3* promoter region if the A allele is present [40]. The GG genotype of rs2232365 was observed to decrease FOXP3 expression, affecting Treg cell function by disruption of the Th1/Th2 balance [40]. Conventionally, Th2-mediated immunity has been considered to favor tumor growth, by promoting angiogenesis as well inhibiting cell-mediated immunity and tumor cell killing [41, 42]. Hence, we inferred that high frequencies of G allele might affect Treg function and decrease Th2 immune response, leading to a protective effect against tumor development.

FOXP3 transcription factor has different expression patterns in a great variety of cell types, and its role in cancer remains unclear. Nowadays, it is well established that this protein can be expressed by different cell types, aside from its expression in Tregs, which include normal [21] and tumor [20, 43] cells. Studies have supported that FOXP3 protein also has different roles, acting as a tumor suppressor protein [21], or as evading mechanisms for tumors, when expressed by Tregs [44]. In breast cancer, the FOXP3 has been described as a transcriptional repressor of genes involved in tumor development, like *HER2* and *SKP2* [21], and also in cancer progression, like *CXCR4* [26].

Notwithstanding, AA homozygous samples for rs3761548 and rs2232365 of *FOXP3* polymorphisms, considered variant and ancestral genotypes, respectively, presented larger tumor size (>8 cm). This could suggest that certain genotypes of *FOXP3* gene might contribute, in some way, to disease prognosis.

In another study [24], the variant genotype AA of *FOXP3* was also positively associated with tumor size, in triple negative breast cancer. Taken together, these results may indicate a role for this marker in cancer progression, raising new possibilities for research, targeting FOXP3.

## Conclusions

In conclusion, the present study demonstrated that *FOXP3* rs2232365 is negatively and *CXCL12* rs1801157 is positively associated with WT susceptibility. Although the number of WT patients in this case-control study was small, the incidence of this cancer is relatively rare in population. Thus, this study demonstrated, for the first time, an association between *FOXP3* and *CXCL12* genetic polymorphisms with this cancer, demonstrating that these markers are, somehow, involved in WT pathogenesis. Further studies are needed to define the precise roles in this process.

## Abbreviations

AS-PCR: Allele-specific PCR
CXCL12: C-X-C chemokine ligand 12
CXCR4: C-X-C chemokine receptor type 4
FOXP3: Forkhead box protein 3
PCR: Polymerase chain reaction
PCR-RFLP: PCR-restriction fragment length polymorphism
SNP: Single nucleotide polymorphisms
Treg: Regulatory T cell
WT: Wilms’ tumor
